# A comprehensive approach to the central monitoring of clinical trials

**DOI:** 10.1186/1745-6215-16-S2-P173

**Published:** 2015-11-16

**Authors:** Julie Bakobaki, Sally Stenning, Nicola Joffe, Sarah Meredith

**Affiliations:** 1 Comprehensive Clinical Trials Unit at UCL, London, UK; 2 MRC Clinical Trials Unit at UCL, London, UK

## Background

As part of a broader methodological programme of work around clinical trial monitoring, we plan to develop a comprehensive central monitoring strategy that is useful to staff developing clinical trial quality management and monitoring plans. In order to facilitate trial operation's staff planning a monitoring strategy where procedural and protocol compliance issues can be identified without the need to perform costly and intensive routine on-site data monitoring, we engaged in the current work that explores practical methods to achieve the aims of trial monitoring and trial conduct oversight.

## Methods

The current work comprises of 3 components: a literature review; informal interviews and surveys of current practice by trial teams at a large UK Clinical Research Collaboration (UKCRC) registered Clinical Trials Unit engaging in phase II and III clinical trials in cancer and HIV research; and strategy development.

## Results

At the time of writing this work is still being conducted. We aim to develop our strategy from the information available from all sources with suggestions for activities centred around: preventing, identifying, monitoring and addressing patient safety and participant rights issues; ensuring, through appropriate trial design and management, the generation of unbiased, accurate and timely data; enhancing trial management processes (including identifying poor performing sites and sites requiring additional support), enhancing protocol compliance, enhancing procedural compliance; and identifying fraudulent activities (fabrication or falsification of data).

